# A network analysis of social problem-solving and anxiety/depression in adolescents

**DOI:** 10.3389/fpsyt.2022.921781

**Published:** 2022-08-10

**Authors:** Qian-Nan Ruan, Ce Chen, De-Guo Jiang, Wen-Jing Yan, Zhang Lin

**Affiliations:** ^1^Wenzhou Seventh People's Hospital, Wenzhou, China; ^2^Department of Psychology, School of Education, Wenzhou University, Wenzhou, China

**Keywords:** network analysis, social problem-solving, anxiety, depression, adolescent

## Abstract

Social problem-solving (SPS) involves the cognitive-behavioral processes through which an individual identifies and copes with everyday problems; it is considered to contribute to anxiety and depression. The Social Problem-Solving Inventory Revised is a popular tool measuring SPS problem orientations and problem-solving styles. Only a negative problem orientation (NPO) is considered strongly related to anxiety and depression. In the present study, we investigated the detailed connections among the five components of SPS and 14 anxiety-depression symptoms and specified the role of NPO and other components in the anxiety-depression network. We employed network analysis, constructed circular and multi-dimensional scaling (MDS) networks, and calculated the network centrality, bridge centrality, and stability of centrality indices. The results were as follows: (1) the MDS network showed a clustering of anxiety and depression symptoms, with NPO and avoidance style components from SPS being close to the anxiety-depression network (demonstrated by large bridge betweenness and bridge closeness); (2) the NPO and positive problem orientation from SPS were most influential on the whole network, though with an opposite effect; (3) strength was the most stable index [correlation stability (CS) coefficient = 0.516] among the centrality indices with case-dropping bootstraps. We also discussed this network from various perspectives and commented on the clinical implications and limitations of this study.

## Introduction

Social problem-solving (SPS) is believed to be strongly related to anxiety and depression, which is very popular among Chinese people. For adults, 4% (1) before and 20.4% (2) during the COVID-19 epidemic suffer from anxiety and depression; for adolescent, the prevalent of anxiety and depression is 11.2%/14.6% (3) before and 19%/36.6% (4) during the epidemic. SPS plays a significant role in psychological adjustment and constitutes an important coping strategy that has the potential to reduce or minimize psychological distress (5, 6). Previous research has found that strong SPS abilities reduce the morbidity associated with anxiety and depression by aiding young people in controlling and modifying their health behavior (7); they are of key importance in managing emotions and wellbeing (8). Conversely, poor problem orientation has consistently linked depression and anxiety (9). Furthermore, depressed patients frequently exhibit deficiencies in social problem-solving, producing fewer effective solutions than do normal control subjects (10).

Essentially, SPS involves the cognitive-behavioral processes through which an individual identifies and copes with everyday problems (11). It comprises problem orientation (a general motivational and appraisal component) and problem-solving style (the cognitive and behavioral activities a person uses to cope with problems). The Social Problem-Solving Inventory Revised (SPSI-R) provides a corresponding scale and comprehensive assessment of all theoretical components linked to contemporary models of social problem-solving [i.e., both problem orientation and problem-solving style (12, 13)]. The SPSI-R consists of a scale of 25 (in the short form) or 52 (in the long form) items, and is one of the most prominent instruments used to study SPS (14). The SPSI-R is a theory-based measure of SPS processes. It consists of five dimensions, as follows: (1) positive problem orientation (PPO), (2) negative problem orientation (NPO), (3) rational PPO problem-solving (RPS), (4) impulsivity/carelessness style (ICS), and (5) avoidance style (AS). The SPSI-R assesses a person's perception of his or her general approach to and styles of solving problems in everyday living that have repeatedly been found to be reliable and valid (15, 16).

SPSI-R research has shown that SPS is an important measure of psychological distress, wellbeing, and social competence [i.e., depression, distress, anxiety, health-related behaviors, life satisfaction, optimism, situational coping, aggression, and externalizing behaviors (17–19)]. Previous research has found that certain specific components of SPS can contribute significantly to anxiety and depression. For example, anxious and depressed patients may have difficulties at different stages of the problem-solving process (20, 21); Kant et al. **(author?)** (22) found that all five problem-solving dimensions measured by the SPSI-R were significantly related to both anxiety and depression in at least one of two samples (i.e., the middle aged and elderly); additional follow-up analyses indicated that NPO contributed most to the significant mediating effect between problems and depression.

Specifically, NPO is strongly related to depression and emotional distress. Abu-Ghazal and Falwah (23) found that employing PPO to solve problems leads to positive psychological wellbeing, while NPO is associated with depression. In Australia, researchers examined the relationship between NPO and depression-anxiety in 285 young adults using the NPO dimensions of the SPSI-R, finding strong connections between the two (24). Additionally, many researchers have found that social anxiety is related to NPO (25, 26). In Hungary, Kasik and Gál (27) studied the relationships among SPS, anxiety, and empathy in 445 Hungarian adolescents, finding that regardless of age, adolescents with an increased level of anxiety also have high levels of NPO and AS. Furthermore, studies have found a link between NPO and stress (28–32). Therefore, anxiety and depression have the strongest association with NPO, above all other SPS components (8, 33–35), and success in reducing symptoms of anxiety and depression appears to be more strongly predicated on the absence of NPO than presence of PPO (34).

These studies suggest that NPO plays an important role in anxiety and depression. We also explored the detailed connections between problem-solving orientations (including NPO) and problem-solving styles with anxiety-depression symptoms. In other words, we integrated the components of SPS into the anxiety-depression network and investigated the link between these components and anxiety-depression symptoms. We identified the components of social problem-solving most strongly associated with certain symptoms in the anxiety-depression network and determined which components were most centrally located.

Thus, network analysis was employed to analyze the relationships among components of SPS and anxiety-depression symptoms, working from the bottom up, without applying any top-down construct consistent with the standard biomedical and reductionist model (36). A key premise of network theory is that psychopathological symptoms are interacting and reinforcing parts of a network, rather than clusters of underlying disorders (37). To test this argument, network analysis has been used to describe the relationships within and between disorders (37). The dynamics and interrelationships between comorbidities can be identified in network analysis and gaps not considered by factor analysis methods can be addressed (38). A network is defined as a set of nodes (symptoms) and edges (connections between nodes). In a network model, the symptoms themselves constitute the disorder. The onset and maintenance of symptoms are determined by tracing the pathways of the network (38).

In an estimated network structure, a centrality measure denotes the overall connectivity of a particular symptom (or component). Central nodes contribute the most to the interrelatedness of symptoms (or components) within the estimated network structure (39, 40). A tightly connected network with many strong connections among the symptoms is considered risky because activation of one symptom can quickly spread to other symptoms, leading to more chronic symptoms over time (41). In other words, when a highly central component is activated (i.e., a person reports the presence of a symptom), it influences other components, causing them to become activated as well, and thus maintaining the network. Considering the importance of problem orientation and problem-solving styles to emotional wellbeing, the nodes should be strongly linked to symptoms of anxiety and depression. In addition, we calculated the bridge-centrality. Previous research has found that deactivating bridge nodes prevents the spread of comorbidity (i.e., one disorder activating another) (42). Through this network analysis, we gained insight regarding the relationship between SPS and anxiety-depression, which may have clinical implications such as helping to modify patients' problem-solving styles to alleviate related symptoms.

In summary, social problem solving is highly correlated with anxiety and depression and can lead to a number of mental illnesses. There are few study about how the aspects of social problem solving that contribute to depression and anxiety and how they both interact with each other. The present study is to explore the detailed connections between problem-solving orientations and problem-solving styles with anxiety-depression symptoms. NPO, specifically, is hypothesized to be related to depression and emotional distress. We characterized the network structure of SPS components and anxiety-depression symptoms using psychiatric and regular samples. We first investigated the node and bridge centrality, and then determined the stability of the centrality indices for the network.

## Methods

### Participants

The samples, consisting of adolescents aged 12–17 years, was obtained from a psychiatric hospital and two secondary schools, collected from October 2021 and completed in March 2022. The 100 adolescents from the hospital were outpatients who had mental health assessments done by psychiatrists. When patients enter the psychological assessment room, they are briefly introduced to the purpose of our study and then asked to fill out the relevant scales based on the most recent week. They could ask the psychiatrists for help if they have any questions. When the task was finished, the psychiatrists have a check to make sure that all responses are completed, and then the subject leaves the assessment room. The other 100 participants were randomly selected middle school students; they conducted the self-rating assessments while monitored by their teachers in the classrooms. All participants signed an informed consent form and were explained about the rules regarding anonymity, confidentiality, and their right to quit.

Ten samples (from the middle schools) were excluded from data collection because they failed the manipulation check (43). Therefore, 190 participants were included in the data analysis.

### Measures

#### Hospital anxiety and depression scale

The HADS assesses both anxiety and depression, which commonly coexist (44). The measure is employed frequently, due to its simplicity, speed, and ease of use. Very few literate people have difficulty completing it. The HADS contains a total of 14 items, including seven for depressive symptoms (i.e., the HADS-D) and seven for anxiety symptoms (i.e., the HADS-A), focusing on symptoms that are non-physical. The correlations between the two subscales vary from 0.40 to 0.74 (with a mean of 0.56). The Cronbach's alpha for the HADS-A varies from 0.68 to 0.93 (with a mean of 0.83) and for the HADS-D from 0.67 to 0.90 (with a mean of 0.82). In most studies, an optimal balance between sensitivity and specificity was achieved when a cut point was set at a score of 8 or above on both the HADS-A and HADS-D. The sensitivity and specificity for both is 0.80. Many studies conducted around the world have confirmed that the measure is valid when used in a community setting or primary care medical practice (45).

#### SPSI-R (Chinese version)

There have been several revised versions of the SPSI-R for use in the Chinese language, such as the measure published by Siu and Shek (46) and Wang (47). The present study used the latter, which shows both good reliability and validity. The overall Cronbach's alpha is 0.85, and the RPS, AS, NPO, PPO, and ICS subscales are 0.85, 0.82, 0.70, 0.66, and 0.69, respectively. The SPSI-R uses a five-point Likert-type scale ranging from 0 to 4, as follows: (0) Not at all true for me, (1) slightly true for me, (2) moderately true for me, (3) very true for me, and (4) extremely true for me.

### Network analysis

We used a Gaussian graphical model (GGM) to build the network *via* the R package (R Core Team version 4.1.3) *qgraph* (version 1.9.2) (48, 49). GGMs estimate many parameters (i.e., 19 nodes required the estimation of 171 parameters: 19 threshold parameters and 19 ^*^ 18/2 = 171 pairwise association parameters) that would likely result in false positive edges. Therefore, it is common to regularize GGMs *via* a graphical lasso (49–51), leading to a sparse (i.e., parsimonious) network that explains the correlation or covariance among nodes with as few edges as necessary. Node placement was determined by the Fruchterman-Reingold (FR) algorithm, which places nodes with stronger average associations closer to the center of the graph (52). The R package *qgraph* was used to calculate and visualize the networks. We also measured the centrality and stability of the established network. The R package *qgraph* and *estimatenetwork* automatically implement the glasso regularization, in combination with an extended Bayesian information criterion (EBIC) model, as described by Foygel and Drton (53).

In network parlance, anxiety-depression symptoms and SPS components are “nodes” and the relationships between the nodes are “edges”. The edge between two nodes represents the regularized partial correlation coefficients, and the thickness of the edge indicates the magnitude of the association. The graphical lasso algorithm makes all edges with small partial correlations shrink to zero, and thus facilitates interpretation and establishment of a stable network, solving traditional lost-power issues that emerge from examining all partial correlations for statistical significance [for greater detail, see (54)]. For the present network, we divided the components into three groups or communities: anxiety (seven symptoms), depression (seven symptoms), and SPS (five components).

**Figure 1:**
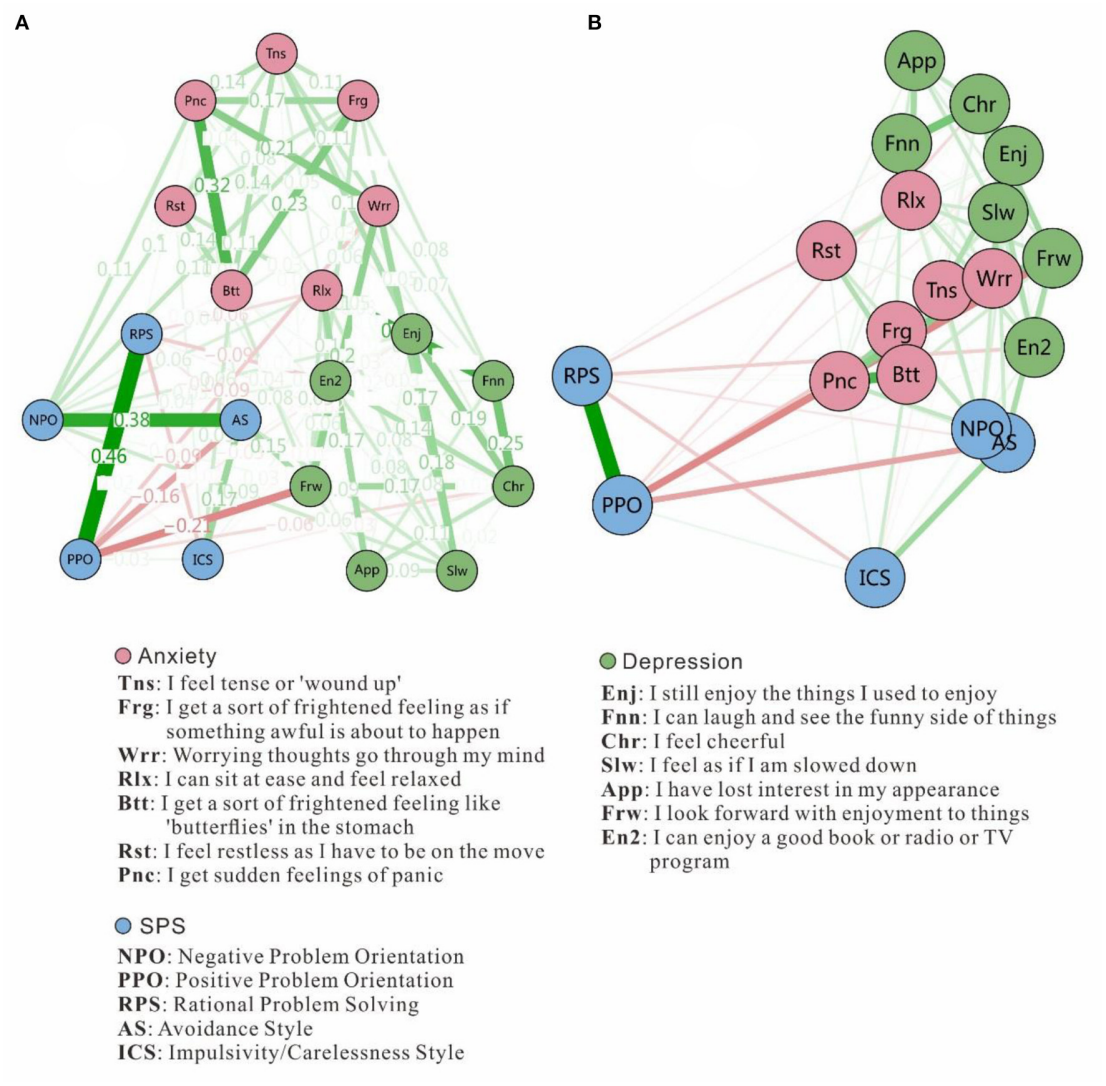
Estimated network structure based on a sample of 190 adolescents. The network structure is a GGM, which is a network of partial correlation coefficients. Green edges represent positive correlations and red edges indicate negative correlations. The thickness of the edge reflects the magnitude of the correlation. **(A)** Network structure with the “circle” layout for easy viewing, but it is important to note that the node positions don't indicate Euclidean distances. **(B)** Network structure with MDS, showing proximities among variables as distances between points in a low-dimensional space.

Most network studies in psychopathology have used the FR algorithm to plot graphs (52). The FR algorithm is a force-directed graph method [see also (55)] that is similar to creating a physical system of balls connected by elastic strings. Importantly, the purpose of plotting with a force-directed algorithm is not to place the nodes in meaningful positions in space, but rather to position them in a manner that allows for easy viewing of the network edges and clustering structures (56). We used the “circle” layout for easier viewing, which places all nodes in a single circle, with each group (or community) put in separate circles (see Figure 1A). In addition, we employed a multi-dimensional scaling (MDS) approach to display the network (see Figure 1B). MDS represents proximities among variables as distances between points in a low-dimensional space [e.g., two or three dimensions; (57)]. MDS is particularly useful for understanding networks because the distances between plotted nodes are interpretable as Euclidean distances (56).

#### Centrality

We calculated several indices of node centrality to identify the symptoms or components most central to the network (58). For each node, we calculated the strength (i.e., the absolute sum of edge weights connected to a node), closeness (i.e., the average distance from the node to all other nodes in the network), betweenness (i.e., the number of times a node lies on the shortest path between two other nodes), and expected influence (i.e., the sum of edge weights connected to a node). For SPS and anxiety-depression networks considering the relationship in both direction (i.e., both positive and negative), strength rather than expected influence (which only calculates neutralized influence) is suitable. The node bridge strength is defined as the sum of the value of all edges connecting a given node in one community with nodes in other communities, and was computed by the R-package *networktools* (42). Higher node bridge strength values indicated a greater increase in the risk of contagion to other groups or communities (42).

#### Stability of centrality indices

We investigated the stability of centrality indices by estimating network models based on subsets of the data and case-dropping bootstraps (*n* = 1,000). If correlation values declined substantially as participants were removed, we considered this centrality metric to be unstable. The robustness of the network was evaluated by the R-package *bootnet* using the bootstrap approach (54). This stability was quantified using the CS coefficient, which quantified the maximum proportion of cases with a 95% certainty that could be dropped to retain a correlation with an original centrality higher than 0.7 (by default) (54).

## Results

The students' average age was 15.54 years (*SD* = 1.302); the group included 102 males and 88 females. We conducted descriptive statistics for the scores of each scale on different demographic variables. The results are shown in Table 1, which demonstrate the number of participants in each group and the mean score and standard deviation (in the parenthesis) for each scale. Due to some missing data for some participants, the total the number of people with different conditions does not equal 190.

**Table 1:** The descriptive statistics of the six SPS components, anxiety, and depression.

		***N***	**RPS *M (SD)***	**AS *M (SD)***	**ICS *M (SD)***	**PPO *M (SD)***	**NPO *M (SD)***	**Anxiety *M (SD)***	**Depression *M (SD)***
Sex	Male	102	43.31 (10.44)	15.20 (6.10)	9.96 (2.85)	13.29 (3.69)	14.54 (5.07)	6.38 (4.66)	6.20 (4.87)
	Female	87	36.25 (11.57)	20.67 (6.63)	10.86 (3.05)	10.09 (3.63)	18.32 (5.08)	10.79 (5.12)	10.23 (5.04)
Family structure	Regular	135	41.65 (11.10)	16.42 (6.34)	10.25 (2.90)	12.58 (3.88)	15.16 (5.02)	7.08 (4.84)	6.66 (4.95)
	Single parent	13	38.77 (13.16)	20.92 (7.26)	10.08 (3.12)	10.08 (3.50)	17.92 (7.01)	11.69 (6.52)	10.38 (5.04)
	Reconstituted	6	36.50 (10.03)	19.33 (8.04)	9.33 (2.88)	9.50 (3.56)	16.50 (6.12)	10.00 (5.80)	9.17 (5.12)
	Orphan	1	26.00	23.00	9.00	10.00	22.00	12.00	12.00
Ranking	Only child	42	42.10 (11.61)	18.12 (6.27)	10.27 (2.83)	12.56 (3.88)	15.10 (5.20)	7.64 (4.44)	7.45 (4.82)
	Eldest child	50	38.33 (11.51)	17.47 (6.97)	10.66 (3.04)	11.62 (4.37)	15.94 (5.42)	8.06 (5.64)	7.28 (5.51)
	Second child	7	37.71 (10.45)	18.71 (5.53)	10.57 (1.81)	10.43 (2.70)	17.00 (6.43)	8.86 (6.12)	10.00 (4.24)
	Youngest child	56	43.14 (10.61)	15.39 (6.42)	9.65 (2.90)	12.76 (3.54)	15.18 (5.15)	7.04 (5.23)	6.32 (4.82)
Economic status	Good	45	39.84 (12.63)	15.57 (6.14)	10.00 (3.10)	12.27 (4.40)	14.31 (4.97)	7.09 (4.79)	6.51 (5.10)
	Normal	101	41.12 (10.68)	17.54 (6.63)	10.23 (2.80)	12.16 (3.68)	16.01 (5.19)	7.73 (5.31)	7.25 (4.95)
	Poor	8	49.38 (7.37)	16.00 (6.63)	10.00 (2.73)	13.63 (3.70)	14.38 (6.95)	7.75 (5.04)	7.38 (5.60)

As for the network, ~41.5% of all 171 network edges were set to zero by the EBICglasso algorithms. Figure 1 presents the network of SPS components and anxiety-depression symptoms. Figure 1A displays an easily viewable circular network with weights on each edge. For example, the strongest edge (weight = 0.32) among the anxiety symptoms was between *Btt*^1^ (“I get sort of a frightened feeling, like 'butterflies' in the stomach”) and *Pnc* (“I get sudden feelings of panic”). Among depression symptoms, the strongest edge (weight = 0.25) was between *Chr* (“I feel cheerful”) and *Fnn* (“I can laugh and see the funny side of things”). For SPS components, the strongest edge (weight = 0.46) was between *PPO* (positive problem orientation) and *RPS* (rational problem-solving). Figure 1B display the MDS network. Highly-related nodes appear close together, whereas weakly-related nodes appear further apart. The anxiety-depression symptoms and SPS components cluster within their own communities, and anxiety-depression nodes are closer to each other. The *NPO* (negative problem orientation) and *AS* (avoidance style) nodes are nearest to the anxiety-depression network, while other components are distant from that network.

### Centrality indices

**Figure 2:**
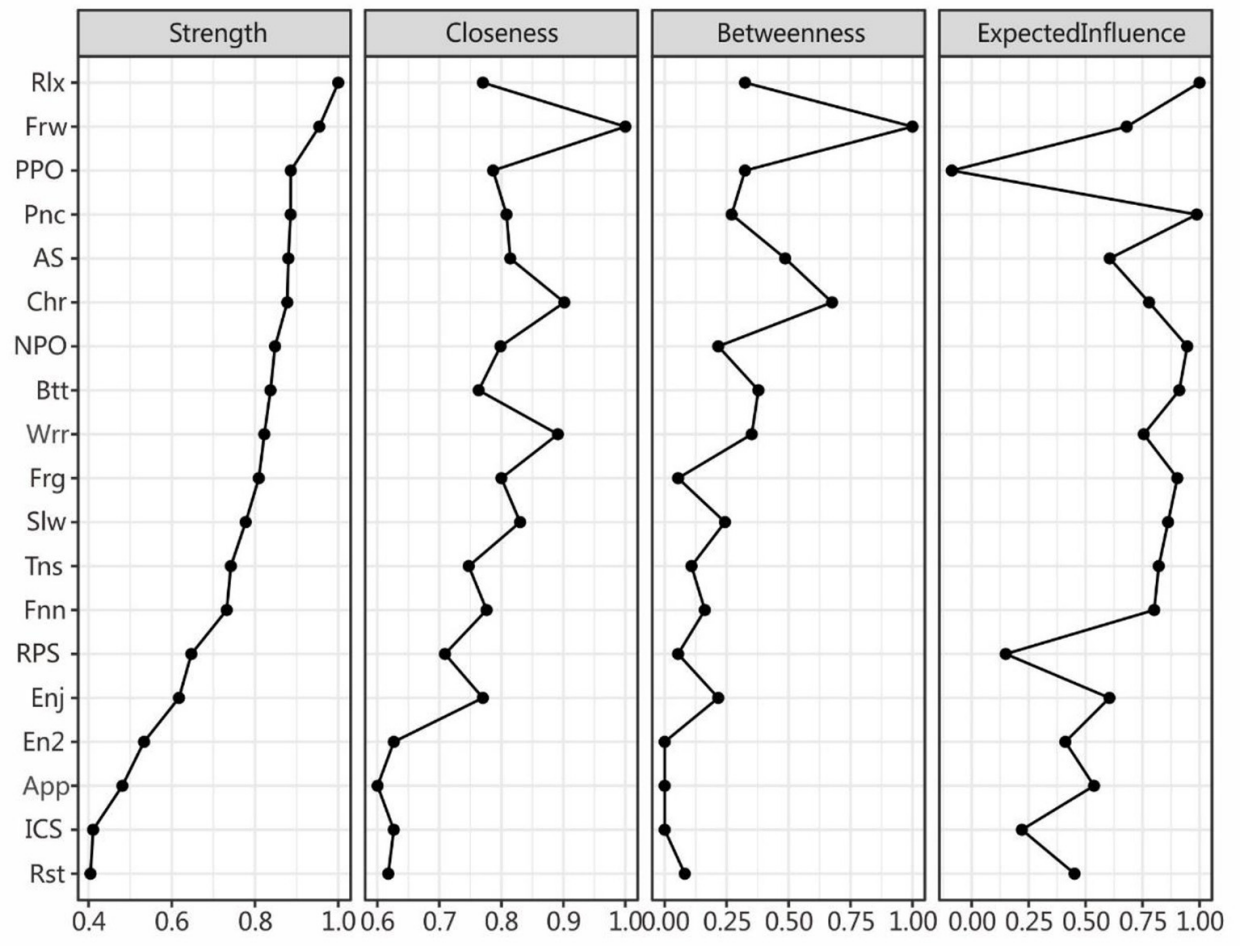
Centrality indices for the nodes of the present network including those for strength betweenness closeness expected influence. The values are normalized to be within the range of 0–1. The full names of the abbreviations can be found in Figure 1.

For the centrality indices, the values were scaled (i.e., normalized) relative to the largest value for each measure. Figure 2 shows the centrality indices, which are ordered by *strength*. For **strength**, *Rlx* (“I can sit at ease and be relaxed”) from the anxiety symptoms is the most central symptom,^2^ followed by *Frw* (“I look forward with enjoyment to things”) from the depression symptoms and *PPO* (positive problem orientation) from the SPS components, indicating that these nodes had the strongest relationships to the other nodes. For **closeness** and **betweenness**, *Frw* again ranked the highest, indicating that it was closest to all other nodes in the network and on the shortest path between two other nodes. As for **expected influence**, considering the direction of the relationship (both positive and negative), *Rlx* and *Pnc* from the anxiety community was most positively and *PPO* most negatively influential on the whole network, indicating that *Rlx* may be an important risk factor and *PPO* an important protective factor. *NPO* most positively influenced the network from the SPS community, and *Slw* (“I feel as if I am slowed down”) did the same for the depression community.

**Figure 3:**
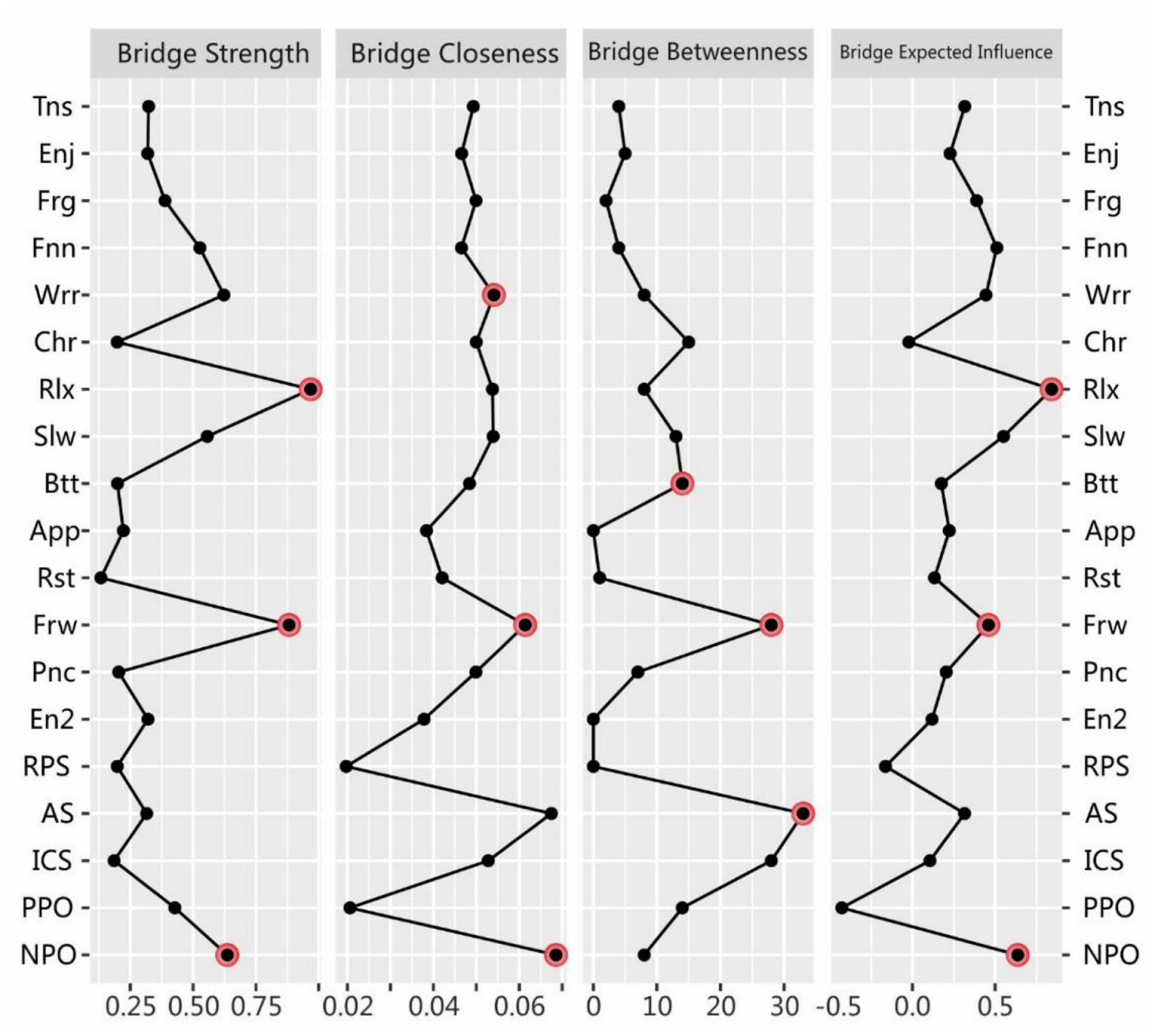
Estimated bridge centrality indices for the present network, including bridge strength, bridge betweenness, bridge closeness, and bridge expected influence. The full names of the abbreviations for the nodes can be found in Figure 1.

We also calculated the bridge centrality indices (see Figure 3). *Rlx, Frw*, and *NPO* for anxiety-depression and SPS were found to have the strongest connections (i.e., bridge strength) with other communities (42). For bridge closeness, *Frw, AS*, and *NPO* ranked the highest. For bridge betweenness, *Frw, AS*, and *ICS* comprised the top three. For bridge expected influence, *Rlx, Slw*, and *NPO* were the most influential.

### Stability of the centrality indices

**Figure 4:**
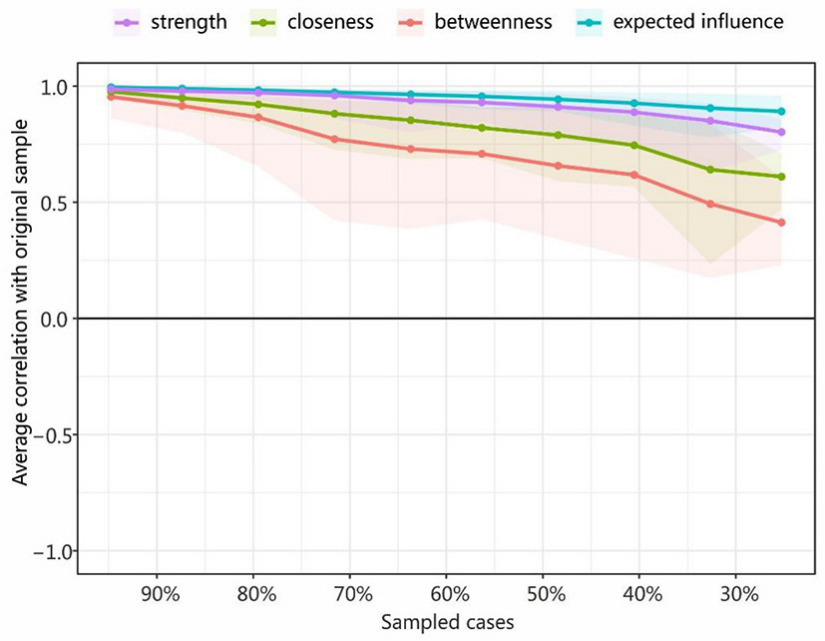
Average correlations between the centrality indices of networks sampled with persons and the original sample. Lines indicate the means and areas ranging from the 2.5th quantile to the 97.5th quantile.

Figure 4 shows that the average correlations dropped between the centrality indices of networks sampled with persons and the original sample. The stability levels of closeness and betweenness dropped steeply, while the stability levels of the node strength and expected influence less so. The Correlation-Stability (CS) coefficient value should preferably be above 0.5 and not be below 0.25 (59). In this research, the CS coefficient indicated that the betweenness [CS (cor = 0.7) = 0.205] was not stable, while the closeness [CS (cor = 0.7) = 0.437] was relatively stable in the subset cases. Node strength and expected influence performed best [CS (cor = 0.7) = 0.516], reaching the cutoff of 0.5 and indicating that the metric was stable. Therefore, we found that the order of node strength and expected influence were most interpretable (with some care), while the order of betweenness was not.

## Discussion

Anchored in the network perspective (39), this study illustrated the node pathways, central indices, and central bridging indices for the SPS and anxiety-depression networks. From a “network-network” perspective, the node connections were closer within (vs. between) the anxiety-depression and SPS networks, demonstrating their relative independence from one other. This result is in keeping with previous comorbidity studies of anxiety and depression that employed network analysis (60, 61), underscoring that the SPS network is distant from the anxiety-depression network (though the *NPO* and *AS* nodes are close to the anxiety-depression network, which can be measured by bridge closeness, as seen in Figure 3). Further, the SPS seems more strongly related to anxiety than depression networks, given the longer mean distance from SPS to depression. The reason could be that anxiety is more related to problems or events (the uncertainty of the future) (62) while depression is more related to self (usually accompanied by low self-esteem, low self-efficacy, and hopelessness) (63). This explanation is reasonable but required further verifications. The MDS structure is a useful tool for displaying the spatial relationships of nodes, and thus its use should be encouraged in the future.

From a “nodes-in-network” perspective, the node centrality indices revealed that the *NPO* node from SPS and *Rlx* and *Frw* from anxiety-depression were likely to be the most central in the entire SPS-anxiety-depression network. Considering that mood disorders affect how people look at and deal with problems, it is appropriate to put anxiety, depression, and SPS components into a single network. In terms of clinical implications, from our results, we can infer that therapy will yield the greatest rewards by modifying *NPO*, encouraging relaxation training, and enhancing the expectation of enjoyment for coming things. In addition, the *NPO* and *AS* nodes are nearest to the anxiety-depression network, especial to the anxiety symptoms. Therefore, we may even consider that *NPO* and AS (very close to each other) are innate components of anxiety, as anxious people are worried about the future but do not positively view the problem and do not actively cope with the problem (64). However, this hypothesis requires further confirmation.

From a “network-node-network” perspective, the results of bridge centrality found that the *NPO* in SPS community had the strongest association (for both bridge strength and bridge closeness) with the anxiety-depression network, echoing previous research that *NPO* most strongly contributes to anxiety and depression. However, *PPO* is located away from the anxiety-depression network and the most negatively correlated (65), as can be seen from the low levels of bridge expected influence and bridge closeness. Furthermore, the *RPS* node is strongly connected with *PPO* but valued low in the four indices of bridge centrality, indicating its unimportance because both of them should “stay away” from the network which is main consists of negative nodes (66). In short, *PPO* is the protective and *NPO* the risk factor for the anxiety-depression network. In clinical settings, encouraging *PPO* and discouraging *NPO* would be an effective approach to reducing symptoms of anxiety and depression.

Some limitations of this research will direct future research. First, a cross-sectional design was adopted to build the SPS and anxiety-depression networks. Therefore, this study could not be used to ascertain whether anxiety-depression symptoms impact SPS components or vice versa. Thus, future work will adopt a longitudinal approach with repeated measures of anxiety-depression and SPS components to clarify the causal relationship between anxiety-depression and SPS components. Second, it is probable that the detected potential pathways among the components are limited to the SPSI-R and HADS scales applied. Self-report tools for the SPSI-R and anxiety-depression usually vary in their constructs. This diversity limits the connections that can be found in terms of network structure. Nevertheless, the scales we used are broadly employed; they were carefully implemented based on their psychometric constructs and applicability for adolescents. Therefore, the present research adds to the literature of how among adolescents, anxiety-depression symptoms may be associated with SPS components. This study may also act as an incentive for future research applying other scales for SPS and anxiety-depression to ascertain the stability of these novel findings.

## Data availability statement

The original contributions presented in the study are included in the article/supplementary material, further inquiries can be directed to the corresponding author.

## Ethics statement

The studies involving human participants were reviewed and approved by Ethics Committee of Wenzhou Seventh People's Hospital. Written informed consent to participate in this study was provided by the participants' legal guardian/next of kin.

## Author contributions

Q-NR conceived and designed the experiments. W-JY and CC performed the experiments. ZL, Q-NR, and W-JY wrote and revised the manuscript. ZL gave financial support. All authors contributed to the article and approved the submitted version.

## Funding

This research was supported by the Medicine and Health Science and Technology Project of Zhejiang, China (No. 2019KY669), and Wenzhou Science and Technology Project of Zhejiang, China (Y20210112).

## Conflict of interest

The authors declare that the research was conducted in the absence of any commercial or financial relationships that could be construed as a potential conflict of interest.

## Publisher's note

All claims expressed in this article are solely those of the authors and do not necessarily represent those of their affiliated organizations, or those of the publisher, the editors and the reviewers. Any product that may be evaluated in this article, or claim that may be made by its manufacturer, is not guaranteed or endorsed by the publisher.

## Footnotes

^1^Following, the node labels with abbreviations will be in italics.

^2^Rlx (“I can sit at ease and be relaxed”) and Frw (“I look forward with enjoyment to things”) are not symptoms *per se*, but for measuring the symptoms “restless” and “pessimistic” using reverse questions.
